# Lung Immune Prognostic Index-Based Predictive Score in Advanced Non-Small Cell Lung Cancer with a Programmed Death Ligand-1 Tumor Proportion Score ≥ 50%

**DOI:** 10.3390/jcm14103543

**Published:** 2025-05-19

**Authors:** Ari Raphael, Ayelet Kamm Feldman, Irina Lazarev, Waleed Kian, Nir Peled, Keren Hod, Walid Shalata, Elizabeth Dudnik

**Affiliations:** 1 Davidoff Cancer Center, Rabin Medical Center, Petah-Tikva 4941492, Israel; 2 Faculty of Medical and Health Sciences, Tel Aviv University, Tel Aviv 6997801, Israel; 3 Goldman School of Medicine, Ben Gurion University of the Negev, Beer Sheva 8410501, Israel; kammayelet@gmail.com (A.K.F.); irinala@assuta.co.il (I.L.); elizabeth.dudnik1603@gmail.com (E.D.); 4 Department of Oncology, Assuta Ashdod Medical Center, Ashdod 7747629, Israel; waleedkian77@gmail.com; 5 Department of Oncology, Shaare Zedek Medical Center, Jerusalem 9103102, Israel; nirp@szmc.org.il; 6 Department of Nutrition Sciences, School of Health Sciences, Ariel University, Ariel 4070000, Israel; hodkeren@gmail.com; 7 Department of Oncology, Soroka Medical Center, Beer Sheva 8410501, Israel; walid.shalata@gmail.com; 8 Thoracic Oncology Department, Assuta Medical Centers, Tel Aviv 6329302, Israel

**Keywords:** biomarkers, lung immune prognostic index (LIPI), advanced non-small cell lung carcinoma (aNSCLC), pembrolizumab, high PD-L1

## Abstract

**Background/Objectives:** The Lung Immune Prognostic Index (LIPI) has emerged as a promising biomarker for predicting outcomes in advanced non-small cell lung cancer (aNSCLC). We assessed whether LIPI, in combination with baseline clinical characteristics, can guide first-line treatment selection between pembrolizumab (P) and pembrolizumab plus platinum-based chemotherapy (PCT) in patients with PD-L1 tumor proportion score (TPS) ≥ 50% and EGFR/ALK/ROS1 wild-type. **Methods:** A predictive score was developed using baseline clinical variables, including age, sex, smoking status, and LIPI, in a proof-of-concept cohort (n = 241). This model was then validated in an independent cohort of 409 patients. OS was compared between patients treated with P versus PCT, stratified by predictive score. **Results:** In the proof-of-concept cohort, the median OS was 18.3 months for P and 26.6 months for PCT (*p* = 0.001). In the validation cohort, the median OS was 28.0 months for P and 22.2 months for PCT (*p* = 0.062). Stratification using the predictive score showed that patients with high scores (3–5) had improved OS with PCT compared to P (31.2 vs. 25.5 months, *p* = 0.001), while those with low scores (0–2) derived similar benefits from both treatments. **Conclusions:** This LIPI-based predictive score may assist in identifying aNSCLC patients who derive greater benefit from chemo-immunotherapy over immunotherapy. Its simplicity and clinical relevance support integration into treatment decision-making, pending prospective validation.

## 1. Introduction

Advanced non-small cell lung cancer (aNSCLC) remains a major global health challenge, accounting for the majority of lung cancer-related deaths worldwide. The emergence of immune checkpoint inhibitors (ICIs) has improved survival in a subset of aNSCLC patients receiving systemic therapy. However, determining which patients are most likely to benefit from immunotherapy remains a central, unresolved issue in the field [1].

While patients with high PD-L1 expression (TPS ≥ 50%) often respond favorably to anti-PD-1/PD-L1 agents, the predictive accuracy of PD-L1 remains limited due to inter-assay variability and intratumoral heterogeneity [2]. Tumor mutational burden (TMB) has also been proposed as a predictor of immunotherapy efficacy, based on the premise that highly mutated tumors may be more immunogenic. However, it remains impractical in routine care due to high costs, complex assays, and inconsistent prognostic data [3,4,5]. 

As alternatives to tissue-based biomarkers, systemic inflammatory markers such as the neutrophil-to-lymphocyte ratio (NLR) and its derivative (dNLR) offer simpler, blood-based surrogates for immune status. Elevated dNLR has been associated with inferior survival outcomes in various cancers, including NSCLC [6]. 

In parallel, serum lactate dehydrogenase (LDH), a marker of tumor burden and metabolic activity, has also shown prognostic value [7,8,9,10]. The Lung Immune Prognostic Index (LIPI), which integrates dNLR > 3 and LDH >upper limit of normal (ULN), has emerged as a composite score to stratify patients into good, intermediate, and poor prognostic groups. Originally validated in retrospective cohorts, LIPI has shown promise in predicting outcomes among ICI-treated patients and may help refine treatment selection strategies [11].

In treatment-naïve aNSCLC, ICIs—either alone or in combination with platinum-based chemotherapy (PCT)—have demonstrated substantial clinical benefits in randomized trials [12]. For patients with PD-L1 TPS ≥ 50%, monotherapy with anti-PD-1/PD-L1 agents is generally favored due to a favorable efficacy-to-toxicity ratio [13]. However, the benefit of combining chemotherapy in this subgroup is unclear, with studies reporting mixed results and concerns about added toxicity [14,15]. Previous attempts to validate dNLR alone as a predictor of ICI efficacy yielded inconsistent results [16]. Building on these findings, the current study investigates whether LIPI can serve as a predictive biomarker—beyond its established prognostic role—in patients with PD-L1 TPS ≥ 50% receiving first-line ICI monotherapy or chemo-immunotherapy.

By evaluating LIPI’s role in guiding treatment intensity, we aim to enable better patient stratification and reduce unnecessary toxicity.

This study is novel in that it proposes a LIPI-based composite predictive score to guide treatment intensity in PD-L1 TPS ≥ 50% aNSCLC patients—a subgroup lacking direct randomized comparisons between monotherapy and combination therapy. To address this, we analyzed outcomes in two independent cohorts: a proof-of-concept cohort (n = 241) and a validation cohort (n = 409), both comprising patients treated with either pembrolizumab monotherapy or chemo-immunotherapy.

## 2. Materials and Methods

### 2.1. Patient Selection

This retrospective study included patients with histologically confirmed advanced-stage non-small cell lung cancer (NSCLC) (stage IV or stage III not amenable to definitive treatment) who were wild-type for *EGFR*, *ALK*, and *ROS1*. Eligible patients had a programmed death ligand 1 (PD-L1) tumor proportion score (TPS) ≥ 50% and received either first-line pembrolizumab monotherapy (P) or pembrolizumab combined with platinum-based chemotherapy (PCT). PD-L1 TPS was assessed by immunohistochemistry (IHC) using the 22C3 pharmDx assay (Agilent Technologies, Santa Clara, CA, USA) [13]. Molecular profiling included real-time polymerase chain reaction (PCR) using the Cobas^®^ (Roche Molecular Systems, Inc., Pleasanton, CA, USA) EGFR test or next-generation sequencing for EGFR mutations, IHC with the D5F3 CDx assay (Roche Diagnostics (Ventana Medical Systems, Inc. (Tucson, AZ, USA))), fluorescent in situ hybridization (FISH (Abbott Molecular Inc. (Des Plaines, IL, USA))), or next-generation sequencing for ALK rearrangements, and FISH or next-generation sequencing for ROS1 rearrangements.

Patients were included if pre-treatment blood test results required to calculate the Lung Immune Prognostic Index (LIPI)—white blood cell count (WBC), absolute neutrophil count (ANC), lactate dehydrogenase (LDH), and the laboratory-specific upper limit of normal (ULN) for LDH—were available within two months prior to treatment initiation. The validation cohort included consecutive eligible patients treated at four Israeli cancer centers (Tel Aviv Sourasky Medical Center, Assuta Medical Center, Ramat Ha-Hayal; Assuta Medical Center, Ashdod; and Shaare Zedek Medical Center, Jerusalem) between June 2016 and December 2020. Patients were excluded if they had a prior invasive malignancy (other than NSCLC) likely to impact overall survival (OS) within five years or were lost to follow-up.

The study was approved by the institutional review boards and ethics committees of all participating centers and was conducted in accordance with the Declaration of Helsinki.

### 2.2. Study Design

We conducted a retrospective analysis using data extracted from electronic medical records. Collected data included baseline demographics, clinical characteristics, pathology reports, treatment details, and laboratory parameters (WBC, ANC, LDH). Patients were categorized into two groups: Group P: Pembrolizumab monotherapy, and Group PCT: Pembrolizumab + platinum-based chemotherapy. OS was the primary outcome. To minimize confounding, propensity score matching (PSM) was performed based on age, sex, and Eastern Cooperative Oncology Group performance status (ECOG PS).

A predictive score was developed using five parameters previously associated with differential outcomes between P and PCT: age ≥ 65 years, female sex, never-smoking status, adenocarcinoma histology, and LIPI ≥ 1 (LIPI was calculated as derived neutrophil-to-lymphocyte ratio [dNLR] > 3 and LDH > ULN). Each variable contributed one point to a cumulative score (range 0–5), with patients stratified into: good risk (0 points), intermediate risk (1 point), and poor risk (≥2 points). For statistical analysis, patients were further grouped into low-risk (score 0–2) and intermediate-high-risk (score 3–5) categories. OS was then compared between treatment groups within these risk categories to assess the predictive value of the LIPI-based score. To validate these findings, OS was reassessed in the independent validation cohort, applying the same inclusion criteria and predictive score methodology. To evaluate the appropriateness of the selected cutoff value of 3, we performed a receiver operating characteristic (ROC) curve analysis in the proof-of-concept cohort. The highest Youden Index was observed at a score threshold of ≥2.5 (Youden Index = 0.198), while a cutoff of ≥3 yielded a comparable Youden Index of 0.156. Both values demonstrated similar discriminatory capacity; however, the threshold of 3 was retained given its balance between statistical performance and clinical interpretability, allowing for stratification into clinically intuitive low-risk (score 0–2) and high-risk (score 3–5) groups. Additionally, multivariable Cox proportional hazards regression, including all five score components (age ≥65, female sex, never-smoking status, adenocarcinoma histology, and LIPI ≥1), revealed consistent directional associations with overall survival, although none reached statistical significance (all *p* > 0.15). These findings support the use of the composite score to enhance treatment selection through the integration of multiple modest but meaningful predictors.

### 2.3. Statistical Analysis

All analyses were performed using SPSS software (Version 29.0.2.0. IBM Corp., Armonk, NY, USA). Categorical variables were summarized as counts and percentages. Continuous variables were reported as medians with interquartile ranges (IQRs) or as means with standard deviations (SDs). OS was analyzed using Kaplan–Meier survival curves, with comparisons via the log-rank test. Follow-up duration was calculated using the reverse Kaplan–Meier method. PSM was used to match patients by age, sex, and ECOG PS. The predictive value of the LIPI-based score was assessed using Kaplan–Meier survival analysis stratified by risk group (low vs. intermediate-high). A two-sided *p*-value < 0.05 was considered statistically significant.

## 3. Results

### 3.1. Baseline, Tumor, and Treatment Characteristics

A total of 241 patients with EGFR/ALK/ROS1 (Thermo Fisher Scientific (Ion Torrent) (Waltham, MA, USA)) wild-type advanced non-small cell lung cancer (aNSCLC) and PD-L1 TPS ≥ 50% were treated with first-line P with or without PCT from June 2016 to December 2020, identified in the proof-of-concept cohort. Additionally, 409 patients from January 2018 to June 2022 were included in the validation cohort.

#### 3.1.1. Proof-of-Concept Cohort

A total of 241 patients were included in the proof-of-concept cohort, of whom 191 (78%) were treated with P and 50 (22%) received PCT [16]. Baseline demographic, clinical, and pathological characteristics are presented in Table 1. The LIPI score was calculated for 186 patients with available blood test results. Among patients in the PCT group, 60% received P with carboplatin/pemetrexed, 28% received P with carboplatin/paclitaxel, 4% were treated with other chemotherapy regimens, and for 8%, the chemotherapy regimen was unknown. Molecular tumor testing was limited. The exact number of PD-L1 TPS-treated patients was available for only 48 (19.9%), and testing for TMB was performed in only 33 patients (13.6%). Therefore, these data were not included in the statistical analyses. 

Due to baseline differences between the groups, a propensity score matching analysis was performed, matching patients for age, sex, and ECOG PS. The matched cohort included 90 patients (45 per treatment group). In this matched group, there were no significant differences in baseline characteristics.

#### 3.1.2. Validation Cohort

The baseline characteristics of the 295 patients (72.1%) who were treated with P and the 114 patients (27.9%) who received PCT are displayed in Table 2. The LIPI score was calculated for 292 patients for whom the blood results required for deriving the score were available. The sex distribution was equal for both treatment groups. Patients treated with P were older (median age 69 years) compared to patients treated with PCT (median age 64 years, *p* < 0.001). As expected, there were fewer patients on the P protocol with ECOG PS 0/1 (66.8%) than those on the PCT protocol. Baseline characteristics are displayed in Table 2.

### 3.2. Overall Survival (OS) in the Entire and Matched Cohorts

**Figure 1:**
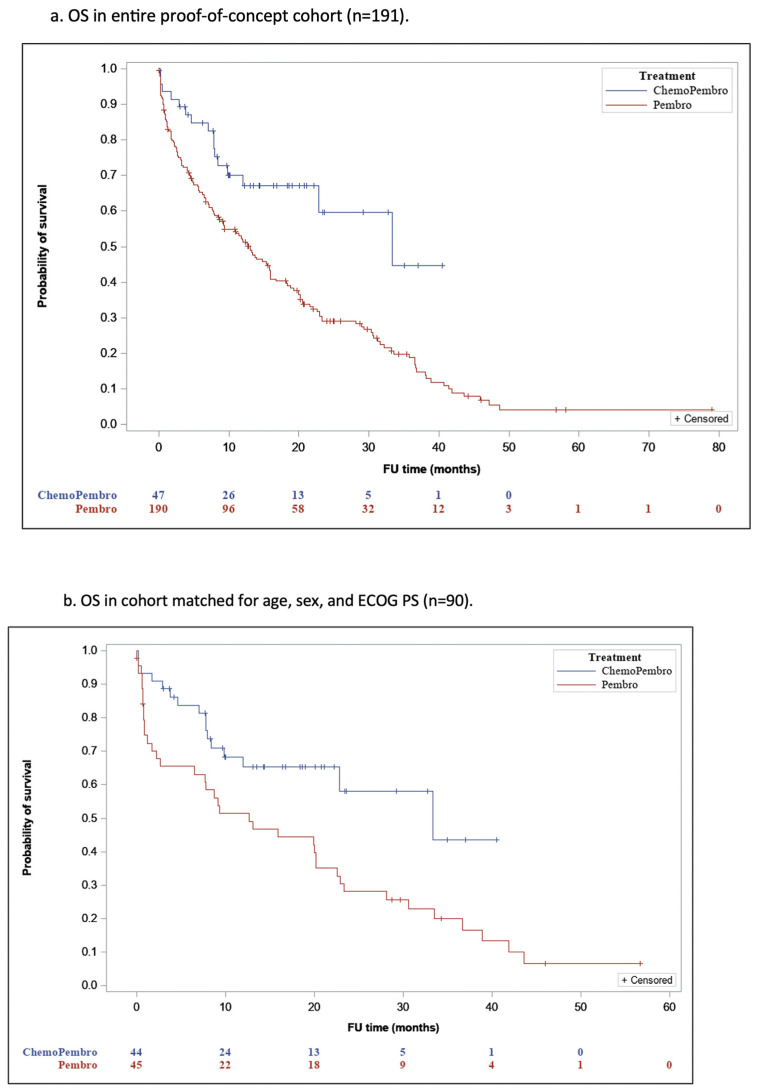

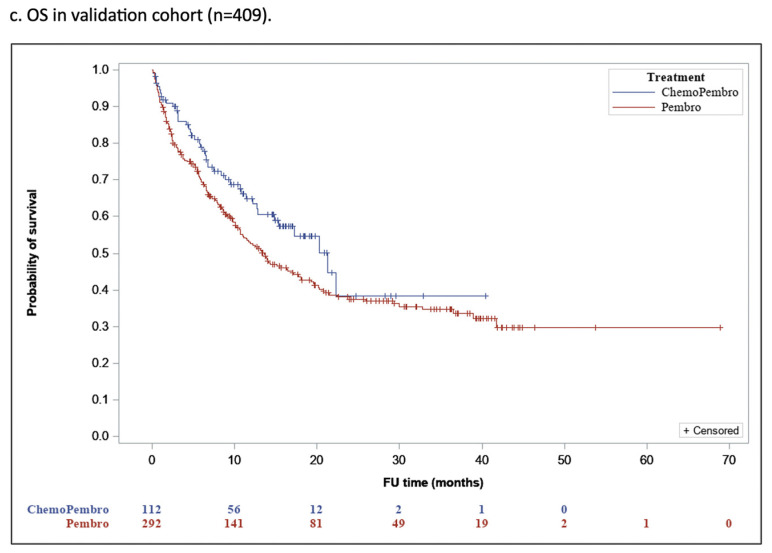
Overall Survival in the Proof-of-Concept Cohort.

The median duration of follow-up was 10.9 months [IQR, 2.6–22.2] for group P and 12 months [IQR, 7–20.8] for group PCT (*p* = 0.42), and 79.6% of patients who received P and 36% of patients who received PCT have died. The median OS (mOS) was 18.3 months (95% confidence interval [CI], 15.3–21.4) in group P and 26.6 months (95% CI, 21.3–32) in group PCT (*p* = 0.001 (Figure 1a).

In the cohort matched for age, sex, and ECOG PS, the mOS was 17.5 months (95% CI, 12.3–22.7) for group P and 26 months (95% CI, 20.6–31.5) for group PCT (*p* = 0.009), reflecting a significantly better OS rate for group PCT compared to group P (Figure 1b).

In the validation cohort, the median follow-up duration was 9.5 months [IQR, 3.3–21.4] for group P and 10.1 months [IQR, 4.5–16.2] for group PCT (*p* =0.36), and 57.3% of the group P patients and 37.7% of the group PCT patients have died. The mOS was 28 months (95% CI, 9.8–17.3) for the former and 22.2 months (95% CI, 13.8–28.7) for the latter (*p* = 0.062) (Figure 1c).

### 3.3. Predictive Score Development and Evaluation

The predictive score was designed to identify patients who may benefit from adding PCT to P in first-line treatment. The score included five predictors that had been shown earlier to predict different outcomes with each therapeutic arm: age ≥ 65 years, female sex, never-smoking status, adenocarcinoma histology, and LIPI ≥ 1. Our predictive score ranges from 0 to 5, with each parameter that is met receiving one point.

**Figure 2:**
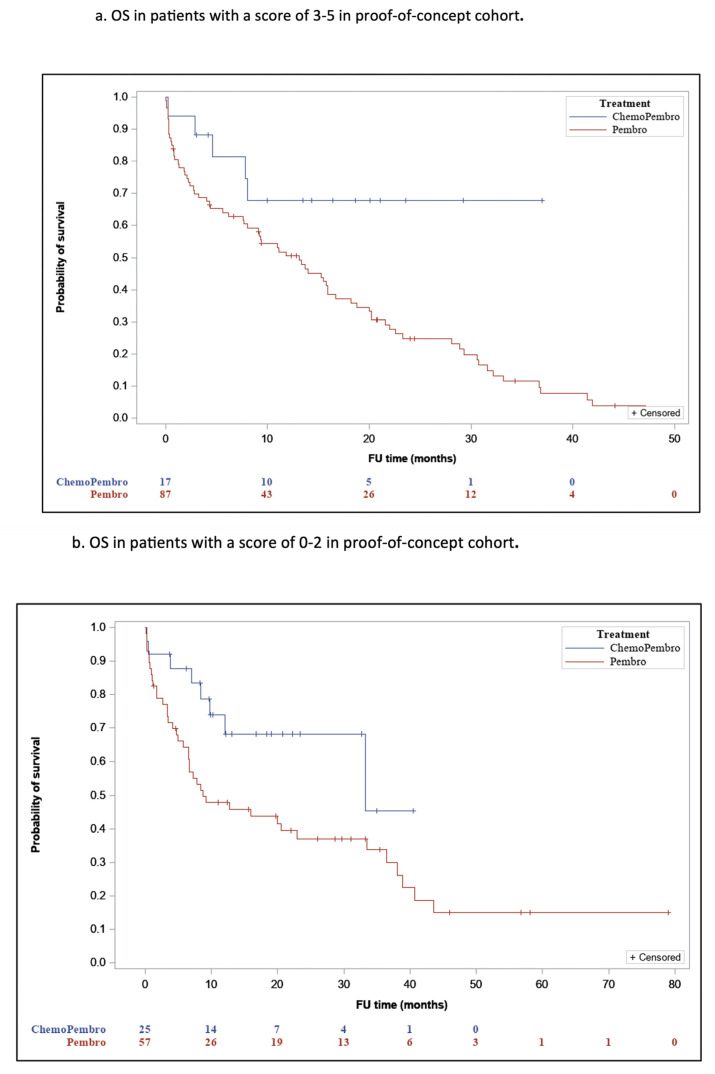

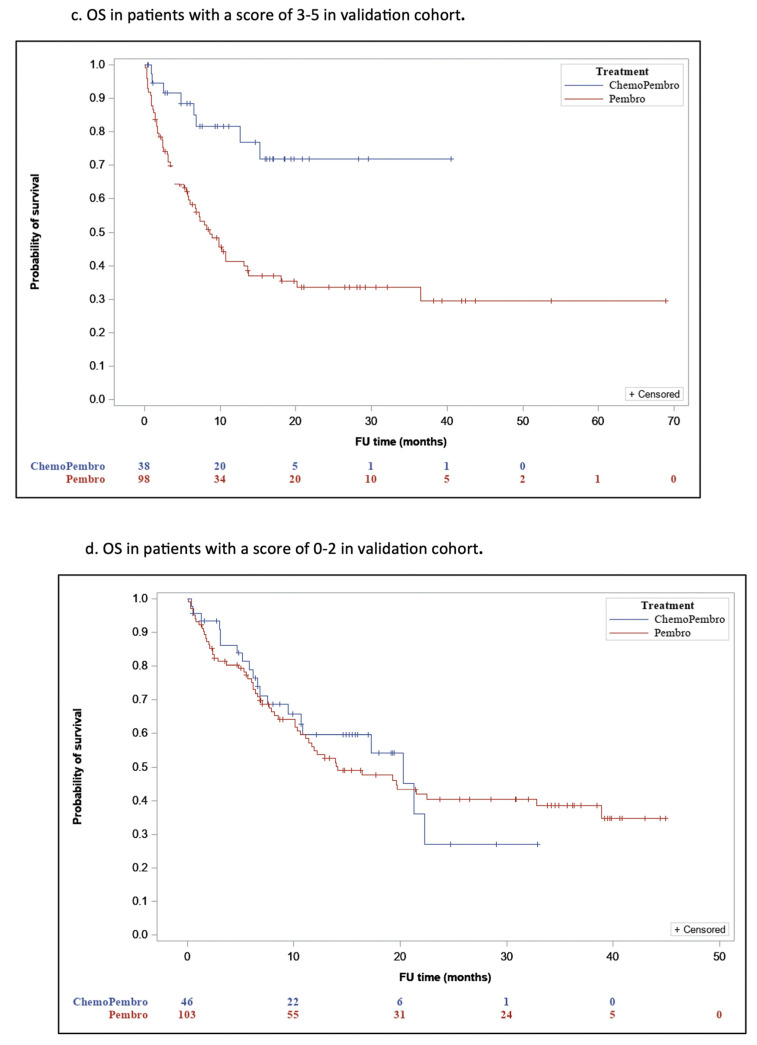
OS According to the LIPI Predictive Score.

The novel LIPI-based predictive score is derived from a previous predictive score created by our group based on dNLR [12] instead of LIPI. Our choice was based upon the well-recognized predictive ability of LIPI as a blood biomarker for ICI monotherapy efficacy, making it the most widely used biomarker in association with ICI treatment. Analysis of the proof-of-concept cohort revealed significant differences in OS between the two treatment groups, both in 0–2 and 3–5 LIPI-based score subsets. For a score of 3–5, the mOS was 15.2 (95% CI, 7.6–18.6) for P and 26.6 (95% CI, NR) for PCT (*p* = 0.03) (Figure 2a), and for a score of 0–2, the LIPI- based score mOS was 24.3 (95% CI, 0–18.7) and 28 (95% CI, NR), respectively (*p* = 0.02) (Figure 2b). A higher numerical difference in OS between P and PCT was observed in the 3–5 LIPI-based score subset. Patients in the validation cohort with a score of 3–5 who were treated with PCT had significantly longer OS (mOS-31.2; 95% CI, NR) compared to patients treated with P (mOS-25.5 months; 95% CI, 5.6–11.6; *p* = 0.001) (Figure 2c). Contrarily, OS was not significantly different between patients with a score 0–2 who were treated with PCT (mOS-18; 95% CI, 10.6–30) and those with a score 0–2 treated with P (mOS-22.5 months; 95% CI, 6.8–21.3; *p* = 0.78) (Figure 2d). The cutoff of ≥3 points was determined based on clinical reasoning and previous prognostic frameworks. Although formal ROC curve analysis was not performed, the threshold was selected to pragmatically differentiate patients most likely to benefit from PCT.

## 4. Discussion

This study used real-world data to compare outcomes of P monotherapy versus P plus PCT in treatment-naïve patients with EGFR/ALK/ROS1 wild-type aNSCLC and PD-L1 TPS ≥ 50%. Our primary aim was to assess whether a Lung Immune Prognostic Index (LIPI)-based predictive score could guide treatment selection by identifying patients who may benefit from the addition of chemotherapy.

We observed different results between the proof-of-concept and validation cohorts. In the proof-of-concept cohort, patients treated with PCT demonstrated significantly improved overall survival (OS) compared to those treated with P alone, both in the entire cohort (median OS [mOS]: 18.3 vs. 26.6 months, *p* = 0.001) and in the propensity score-matched cohort (mOS: 17.5 vs. 26 months, *p* = 0.009). However, this survival advantage was not observed in the validation cohort, where mOS was 28 months in the P group and 22.2 months in the PCT group (*p* = 0.062). These divergent results between the proof-of-concept and validation cohorts may be partially explained by differences in clinical practice patterns across institutions. As this was a retrospective, multicenter study based on two independently collected cohorts, variations in treatment decisions could reflect center-specific preferences. Importantly, no changes occurred in guidelines, treatment paradigms, or patient eligibility criteria during the study period. These conflicting findings align with previous clinical trials and meta-analyses. The KEYNOTE-024 and KEYNOTE-042 trials reported 3-year OS rates of 31–44% for P monotherapy in patients with PD-L1 TPS ≥ 50% [17,18], whereas PCT yielded a 3-year OS of 44% in similar patients [19]. Several meta-analyses found no OS advantage for the addition of chemotherapy to P in patients with high PD-L1 expression [20,21]. Furthermore, real-world studies—including data published by our group—have also supported P monotherapy as the preferred approach for patients with PD-L1 TPS ≥ 50% [22,23,24].

Despite this, the role of chemotherapy in this setting remains controversial. In clinical practice, PCT is often chosen for patients who present with a high tumor burden, rapid disease progression, or significant symptoms, despite high PD-L1 expression. However, given the absence of direct randomized comparisons between P and PCT in this population, additional tools—such as the LIPI-based predictive score—may provide a more personalized treatment approach.

Our predictive score was designed to determine which patients would derive the most benefit from adding PCT to P. The score incorporated five key factors: age ≥ 65 years, female sex, never-smoking status, adenocarcinoma histology, and LIPI ≥ 1.

While the proof-of-concept cohort did not demonstrate a significant predictive ability, we observed a trend favoring improved OS in patients with higher scores. Therefore, we applied the score to the validation cohort (which included twice as many patients as the proof-of-concept cohort) and found a significant association between the score and OS. Patients with a score of 3–5 had significantly improved survival with PCT (mOS: 31.2 months) compared to P (mOS: 25.5 months, *p* = 0.001), while patients with a score of 0–2 had no significant survival difference between PCT (mOS: 18 months) and P (mOS: 22.5 months, *p* = 0.78). These findings are consistent with previous studies on dNLR-based predictive scores, which also showed a positive correlation between OS and PCT treatment in patients with high scores [16].

LIPI is a well-established biomarker for predicting outcomes in patients treated with immune checkpoint inhibitors (ICIs) [25,26]. Poor pretreatment LIPI has been correlated with worse survival in patients receiving ICI monotherapy, but not with chemotherapy outcomes [11]. Our findings support the hypothesis that patients with a poor LIPI score might benefit more from the addition of PCT to P. Potential biological explanations for the prognostic power of LIPI include the association of high dNLR with a systemic inflammatory response, promoting tumor progression and immune suppression, and elevated LDH reflecting increased tumor burden and a hypoxic tumor microenvironment, both of which are associated with inferior immunotherapy outcomes [11].

To validate these findings, further prospective studies in larger cohorts are necessary.

This study has several limitations. The small sample size in the proof-of-concept cohort, particularly in the PCT group, limited statistical power. To mitigate baseline imbalances and reduce potential selection bias, propensity score matching was applied. In the validation cohort, which was larger and used exclusively for model testing, we did not repeat individual subgroup analyses by clinical variables. The retrospective study design introduces potential selection and information biases. In real-world settings, the choice between P and PCT is typically guided by physician judgment and patient-specific clinical characteristics. There is often a bias toward selecting PCT in patients with more aggressive disease presentations, younger age, and better functional status. Consequently, such selection bias may have contributed to differential survival outcomes observed across treatment groups. Additionally, shorter follow-up in the PCT group may have influenced OS comparisons. Due to limited molecular testing data, we could not evaluate correlations with PD-L1 TPS numerical values or tumor mutational burden (TMB). Another limitation is that PD-L1 expression was assessed locally at each participating center without a centralized pathology review, which may have introduced variability in PD-L1 scoring. In addition, in the proof-of-concept cohort, the date of death was unknown for 44 patients, necessitating the use of the last follow-up dates for OS analysis. Finally, the study population was restricted to Israeli centers, potentially limiting the generalizability of the findings across different geographic and ethnic backgrounds.

Given the promising results of the LIPI-based predictive score in the validation cohort, future research should focus on prospective validation in larger, multicenter cohorts, exploring biological mechanisms linking LIPI to immunotherapy outcomes, assessing additional inflammatory markers in combination with LIPI for improved predictive accuracy, and incorporating molecular biomarkers such as TMB and PD-L1 expression levels to refine patient selection.

Furthermore, LIPI scoring is based on routine, low-cost laboratory tests (complete blood count and LDH), making it an economically feasible tool to implement broadly without imposing additional financial burden on healthcare systems.

## 5. Conclusions

This study underscores the potential of a LIPI-based predictive score in guiding first-line treatment decisions for patients with PD-L1 TPS ≥ 50% aNSCLC. While P monotherapy remains the preferred treatment for most patients, adding chemotherapy may improve survival in specific subgroups. Patients with low LIPI scores (0 points) may be treated with P alone, avoiding unnecessary chemotherapy-related toxicity and preserving quality of life. Patients with high LIPI scores (3–5 points) demonstrated a significant survival benefit when treated with PCT instead of P alone. These findings emphasize the importance of personalized treatment approaches that integrate clinical, tumor, and inflammatory biomarkers to optimize patient outcomes. By incorporating LIPI scoring into shared decision-making, clinicians can balance survival benefits with treatment convenience, toxicity, and patient preferences. Given the promising results in the validation cohort, further prospective validation in larger, multicenter studies is warranted to confirm the LIPI-based predictive score as a clinical decision-making tool in advanced NSCLC.

## Figures and Tables

**Table 1:** Baseline and treatment characteristics of patients in the proof-of-concept cohort.

	Entire Cohort (n = 241)			Patients Matched for Age, Sex, ECOG PS (n = 90)		
	Patients Treated with P (n = 191)	Patients Treated with PCT (n = 50)	*p* Value	Patients Treated with P (n = 45)	Patients Treated with PCT (n = 45)	*p* Value
Age, years—mean (SD)	70.4 (10)	64.7 (7.6)	0.00	66.5 (6.9)	65.5 (9.8)	0.37
Sex, n (%)			1.00			1.00
Female	70 (36.6)	18 (36)		17 (37.8)	17 (37.8)	
Male	121 (63.4)	32 (64)		28 (62.2)	28 (62.2)	
Smoking history, n (%)			0.06			0.28
Current/past smoker	140 (73.3)	43 (86)		34 (75.6)	39 (86.7)	
Never smoker	51 (26.7)	7 (14)		11 (24.4)	6 (13.3)	
Histological subtype, n (%)			0.39			0.32
Adenocarcinoma	141 (73.8)	40 (80)		31 (68.9)	37 (82.2)	
Squamous cell	40 (20.9)	6 (12)		11 (24.4)	5 (11.1)	
NSCLC NOS/other	10 (5.3)	4 (8)		3 (6.6)	3 (6.6)	
Stage, n (%)						
IV	189 (99)	48 (96)		45 (100)	43 (95)	
NA	2 (1)	2 (4)		0 (0)	2 (5)	
ECOG PS at diagnosis, n (%)			0.01			1.00
0/1	138 (72.2)	44 (88)		39 (86.6)	39 (86.6)	
2/3/4/5	52 (27.3)	6 (12)		6 (13.3)	6 (13.3)	
NA	1 (0.5)	0 (0)		0 (0)	0 (0)	
Brain metastases, n (%)			1.00			0.30
Yes	45 (23.6)	11 (22)		12 (26.7)	10 (22.2)	
No	146 (76.4)	38 (76)		33 (73.3)	34 (75.6)	
NA	0 (0)	1 (2)		0 (0)	1 (2.2)	
Liver metastases, n (%)			0.41			0.77
Yes	40 (20.9)	7 (11)		11 (24.4)	6 (13.3)	
No	151 (79.1)	42 (89)		34 (75.6)	38 (84.4)	
NA	0 (0)	1 (2)		0 (0)	1 (2.2)	
LIPI, n (%)						
0	39 (20.4)	18 (36)		5 (11.1)	15 (33.3)	
1	59 (30.9)	18 (36)		16 (35.6)	16 (35.6)	
2	47 (24.6)	8 (16)	0.10	21 (46.7)	8 (17.8)	0.00
½	106 (55.5)	26 (52)		37 (82.2)	24 (53.3)	
NA	46 (24.1)	6 (12)		3 (6.7)	6 (13.3)	

**Table 2:** Baseline and treatment characteristics of patients in the validation cohort.

	Entire Cohort (n = 409)		
	Patients Treated with P (n = 295)	Patients Treated with PCT (n = 114)	*p* Value
Age, years—mean (SD)	69.2 (10.3)	64.3 (9.2)	0.00
Sex, n (%)			0.48
Female	100 (33.9)	43 (37.7)	
Male	195 (61.1)	71 (62.3)	
Smoking history, n (%)			0.06
Current/past smoker	263 (89.2)	102 (89.5)	
Never smoker	30 (10.2)	11 (9.6)	
NA	2 (0.7)	1 (0.9)	
Histological subtype, n (%)			0.39
Adenocarcinoma	222 (75.3)	74 (64.9)	
Squamous cell	50 (16.9)	27 (23.7)	
NSCLC NOS/other	23 (7.8)	12 (10.5)	
NA	0 (0)	1 (0.9)	
Stage, n (%)			1.00
IV	277 (93.9)	106 (93)	
III (not amenable to definitive treatment)	10 (3.4)	3 (2.6)	
NA	8 (2.7)	5 (4.4)	
ECOG PS at diagnosis, n (%)			0.223
0/1	197 (66.8)	89 (78)	
2/3/4/5	87 (29.5)	22 (19.3)	
NA	11 (3.7)	3 (2.6)	
Brain metastases, n (%)			0.53
Yes	82 (27.8)	28 (24.6)	
No	213 (72.2)	86 (75.4)	
Liver metastases, n (%)			0.61
Yes	35 (11.9)	16 (14)	
No	260 (88.1)	98 (86)	
LIPI, n (%)			
0 (Good)	81 (27.5)	29 (25.4)	
1 (Intermediate)	80 (27.1)	43 (37.7)	
2 (Poor)	45 (15.3)	14 (12.3)	0.24
½	161 (54.6)	72 (63.1)	
NA	89 (30.1)	28 (24.6)	

## Data Availability

All materials, data, and protocols used in this study are available upon request.

## Abbreviations

The following abbreviations are used in this manuscript:

aNSCLC	Advanced Non-Small Cell Lung Cancer
ALK	Anaplastic Lymphoma Kinase Gene
CI	Confidence Interval
ECOG PS	Eastern Cooperative Oncology Group Performance Status Score
EGFR	Epidermal Growth Factor Receptor Gene
IQR	Inter-Quartile Ranges
LIPI	Lung Immune Prognostic Index
mOS	Median Overall Survival
NA	Not Specified/Not Available
NOS	Not Otherwise Specified
NR	Not Reached
P	Pembrolizumab
PCT	Combination of Pembrolizumab with Platinum-Based Chemotherapy
PD-L1	Programmed Death-Ligand 1
Pts	Patients
PSM	Propensity score matching
ROS1	Proto-Oncogene Tyrosine-Protein Kinase ROS1
SD	Standard Deviation
TPS	Tumor Proportion Score
